# Two-Week Repeated Oral Dose Toxicity Study of Mantidis Ootheca Water Extract in C57BL/6 Mice

**DOI:** 10.1155/2019/6180236

**Published:** 2019-04-07

**Authors:** Hye-Sun Lim, Yun Soo Seo, Seung Mok Ryu, Byeong Cheol Moon, Goya Choi, Joong-Sun Kim

**Affiliations:** Herbal Medicine Resources Research Center, Korea Institute of Oriental Medicine, 111Geonjae-ro, Naju-si, Jeollanam-do 58245, Republic of Korea

## Abstract

*Background. *Mantidis Ootheca (MO), described as the ootheca of* Hierodula patellifera* Serville, 1839,* Tenodera angustipennis* (Saussure, 1869), or* Statilia maculate* (Thunberg, 1784) in Korean Herbal Pharmacopoeia, is an important herbal material that has been traditionally used for treating several medical conditions including renal failure, spermatorrhea, and pediatric enuresis in Korea.* Objective. *The present study investigated the potential subacute toxicity of MO water extract during a 2-week repeated oral administration of doses of 0, 50, 150, or 450 mg/kg/day to C57BL/6 male mice by gavage.* Methods. *The following parameters were examined during the study period: mortality, clinical signs, body weight, hematology, serum biochemistry, gross findings, organ weight, and histopathology. All the mice were euthanized at the end of the treatment period.* Results.* No treatment-related changes in mortalities, clinical signs, body weight, gross finding, and organ weight change were detected after 14 days of oral MO extract administration. In addition, no meaningful MO extract treatment-related changes were observed in the hematological, serum biochemical, and histopathological parameters compared with the normal control group following treatment with doses of up to 450 mg/kg/day.* Conclusion.* Based on these findings, we concluded that treatment of mice with the water extract of MO did not result in significant toxicity and, therefore, it could be considered safe for further pharmacological studies.

## 1. Introduction

Insects have been commonly used as a source of food and drug resources worldwide [1]. Animal-based therapeuticals including insects have remained a component of traditional medicine and are increasingly gaining interest and attention as part of animal-based medicine [2]. Korean history provides the most well-known historical record of animal-based medicinal in the medical book entitled “Donguibogam” written by Heo Jun, an Eastern physician (1546–1615). Dr. Heo recorded the use of 95 different varieties of animal-based medicines including insects for the effective treatment of certain illnesses [3]. For example, grasshoppers have been used to heal bronchitis and asthma, and crickets have been used to alleviate symptoms related to liver diseases and fever [4]. Recently, the focus of drug-related research has shifted to insect-based products with a potential to be used as medicines to treat a variety of diseases. In this regard, several insect varieties to species have been tested by pharmaceutical companies as potential sources of modern drugs [5]. However, despite the increasing popularity of insect-based medicines, limited research is available on the safety of these products. Therefore, toxicological assessment of insect-based drugs is crucial for safe use and regulation of doses to achieve maximum benefits.

**Figure 1:**
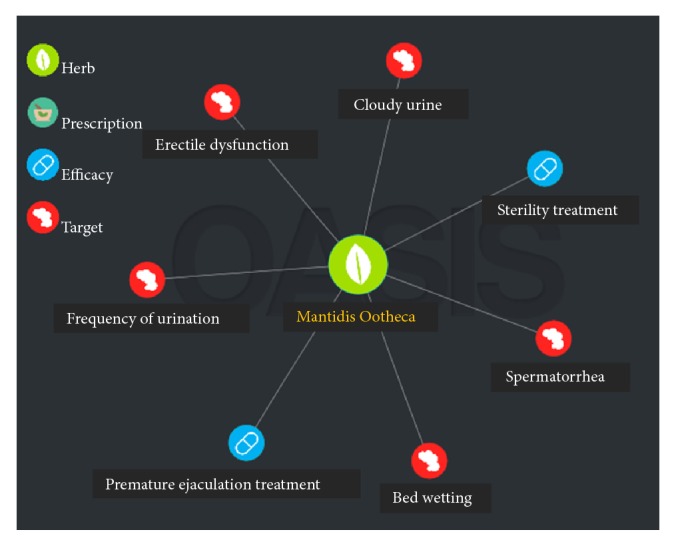
Oriental Medicine Advanced Searching Integrated System (OASIS) analysis of MO.

The authentic Mantidis Ootheca (MO) refers to the dried ootheca of* Hierodula patellifera* Serville, 1839,* Tenodera angustipennis* (Saussure, 1869), and* Statilia maculate* (Thunberg, 1784) which belongs to the insects of the Mantidae family in Korea. It has been widely used as a traditional medicine to treat kidney-related complications such as frequent urination, incontinence, and cloudy urine [6]. In addition, results from the electronic databases research related to MO using the Oriental Medicine Advanced Searching Integrated System (OASIS) reveal MO to have been used as an effective drug in treating sterility and premature ejaculation (Figure 1). Pharmacological studies reported that MO increased the index of testis and thymus gland, and exerted an antidiuretic effect, and decreased lipid peroxidation in the liver of hypercholesterolemia rats [7]. Despite these studies and the widespread use of MO in traditional medicine, no study on its toxicological profile has been reported. With this background, we investigated the possible toxic effects of oral administration of MO water extract in mice in a 2-week study.

## 2. Materials and Methods

### 2.1. Preparation of MO Extract

MO was purchased from a medicinal herb shop in Kwong Mungdang (Ulsan, Korea) in June 2017 and was authenticated by Dr. Goya Choi (Herbal Medicine Resources Research Center, Korea Institute of Oriental Medicine, Naju, Korea). A voucher specimen (accession number: 2-18-0123) was deposited at the Herbal Medicine Resources Research Center, Korea Institute of Oriental Medicine. MO (2.0 kg) was extracted in distilled water (5 × 5 L) for 3 h under reflux (100 ± 2°C). After filtrating and evaporating the solvent* in vacuo*, the remaining liquid was removed by freeze drying to obtain a distilled water-soluble powder extract (23.6 g).

### 2.2. Experimental Animals

**Figure 2:**
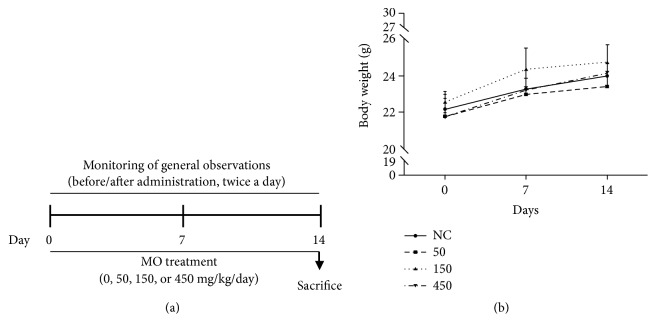
Toxicological evaluation of MO extract in mice. (a) Schematic diagram of drug treatment, tissue preparation, and toxicological evaluation. (b) Mean body weights of mice exposed to repeated doses of 0, 50, 150, and 450 mg/kg/day of MO extract. Values are expressed as means ± SD. SD, standard deviation.

Twenty, specific pathogen-free, 7-week-old C57BL6 male mice (20 ± 2 g) were purchased from DooYeol Biotech (Seocho-gu, Seoul, Korea). After 1 week of quarantine and acclimatization to the animal care facility, mice were randomly divided into four groups (n = 5 per group): one normal control (0 mg/kg/day) and three MO extract groups (50, 150, and 450 mg/kg/day). The experiment design is outlined in Figure 2(a). All mice were housed in a room maintained at a temperature of 23 ± 3°C with a relative humidity of 50 ± 10%, an air ventilation frequency of 10 to 20 times/h, and a light intensity of 150 to 300 Lux with artificial lighting from 08:00 to 20:00. The animals were kept in stainless wire cages and fed a commercial pellet diet (Nestlé Purina Pet Care Company; Bundang-gu, Seongnam, Korea) and sterilized tap water was provided ad libitum. All experimental procedures were conducted in accordance with the National Institutes of Health (NIH) Guidelines for the Care and Use of Laboratory Animals. The study was approved by the Institutes of Animal Care and Use Committee of the Korean Institute of Oriental Medicine (Approval number: 18-041). Animal handling was performed in accordance with the dictates of the National Animal Welfare Law of Korea.

### 2.3. General Observations

Characteristics such as mortality, clinical signs, and body weight of mice were monitored for 14 days. Mortality and clinical signs were recorded twice a day (before and after administration) during the study period. The body weight of each mouse was measured at the initiation of administration of the extract and once a week throughout the study period.

### 2.4. Necropsy

At the end of experiments, all animals that survived were anesthetized using ethyl ether and euthanized by exsanguination via the aorta. Complete gross postmortem examinations were performed with special attention to all vital organs and tissues. Absolute organ weights were measured, and relative organ weights were calculated for the lungs, liver, spleen, kidneys, and testis.

### 2.5. Histopathology

To perform histopathology, the liver tissue was fixed in 10% neutral-buffered formalin. Tissue samples were then embedded into paraffin and sectioned into 4 *μ*m thick slices, followed by staining with hematoxylin and eosin (H&E) solution (Sigma-Aldrich, St. Louis, MO, USA). The stained sections were examined under a light microscope (Olympus Microscope System CKX53; Olympus, Tokyo, Japan).

### 2.6. Hematology

Blood samples from the experimental mice were collected into complete blood count bottles containing ethylenediaminetetraacetic acid (EDTA)-2K (Sewon Medical Co., Cheonan, Korea), and were analyzed for red blood cell (RBC) count, hemoglobin (HB) concentration, hematocrit (HCT), mean corpuscular volume (MCV), mean corpuscular hemoglobin (MCH), mean corpuscular hemoglobin concentration (MCHC), platelet (PLT), white blood cell (WBC) count, and differential leucocyte count (neutrophils, lymphocytes, monocytes, eosinophils, and basophils) using an ADVIA 2120i hematology analyzer (Siemens; Tarrytown, NY, USA).

### 2.7. Serum Biochemistry

For assessing biochemical parameters, blood samples from the experimental mice were centrifuged at 3,000 rpm for 10 min in a separation tube on the day of necropsy and analyzed using a TBA 120FR chemistry analyzer (Toshiba Co., Tokyo, Japan). The serum biochemical parameters measured were creatinine (CRE), glucose (GLU), glutamic oxaloacetic transaminase (GOT), glutamic pyruvic transaminase (GPT), blood urea nitrogen (BUN), and total bilirubin (TBIL).

### 2.8. Statistical Analyses

Body weight, hematology, serum biochemistry, and organ weight values are presented as mean ± standard deviation (SD). The statistical significance between the groups was analyzed using an analysis of variance (ANOVA), followed by Dunnett's multiple comparison test. We did not calculate the median lethal dose (LD_50_) because no mortality was observed.

## 3. Results

### 3.1. Clinical Signs and Change of Body Weight in Mice Treated with MO Extract

In the present study, we did not observe any mortality in mice administered the oral extract of MO during the 2-week study period (Table 1). In addition, as shown in Table 2, no MO treatment-related clinical signs were observed in mice during the study period. At the scheduled necropsy, no gross findings in mice were observed either. A time- and dose-dependent increase in the body weight of mice was observed (Figure 2(b)). There were no statistically significant changes in body weight between the 450 mg/kg/day group and the normal control group (0 mg/kg/day).

### 3.2. Gross Findings in Mice Treated with MO Extract

Necropsy was performed 2 weeks after the administration of the MO extract. We did not observe any abnormal finding in the internal organs including the lungs, liver, spleen, kidneys, and testis (Table 3).

### 3.3. Organ Weight and Histopathological Changes in Mice Treated with MO Extract

**Figure 3:**
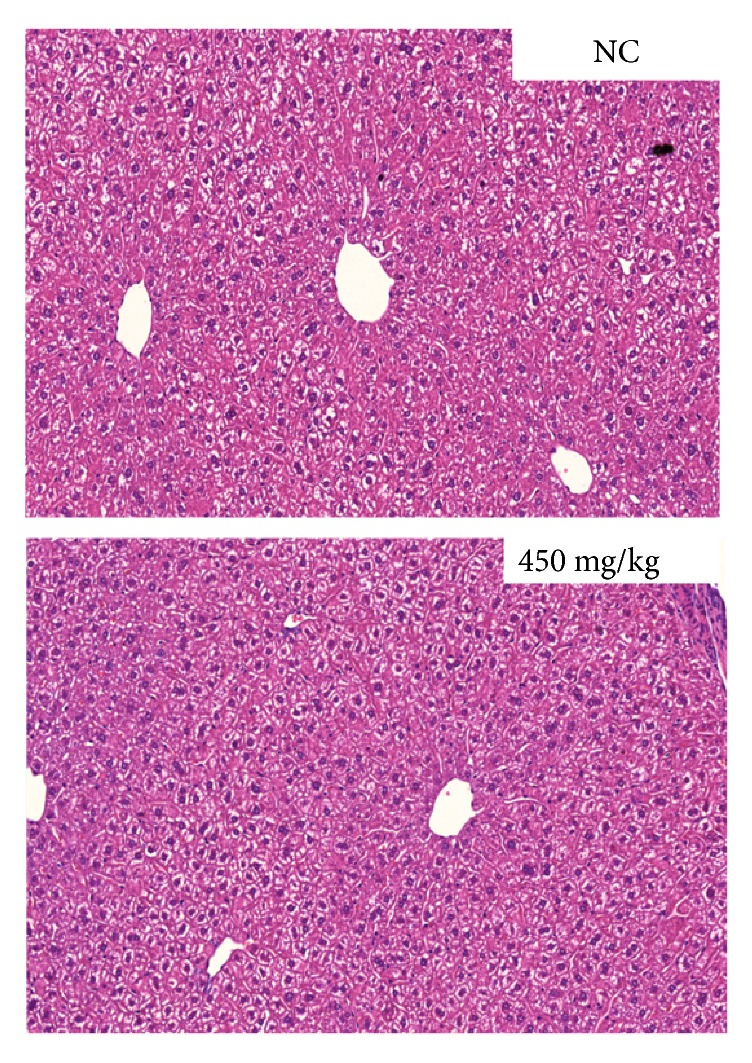
Histopathological examination of the liver in mice exposed to repeated MO extract doses of 0 and 450 mg/kg/day. Representative images (H&E stained) showing histological changes in the liver of vehicle and 450 mg/kg/day-treated mice samples.

We next monitored changes in organ weight and histopathological changes in mice treated with MO extract. Mice in the MO extract-treated groups showed no significant change in the absolute weight of the five principal organs, namely, the lungs, liver, spleen, kidneys, and testis compared with organs in the normal control group (Table 4). Similarly, histopathological examination of the liver revealed no abnormal findings at the highest dose of 450 mg/kg/day in the MO extract-treated group compared with the normal control group (Figure 3).

### 3.4. Hematology and Serum Biochemistry in Mice Treated with MO Extract

Table 5 shows the results of the hematological analyses. No significant differences were observed in the hematological parameters of the MO extract-treated mice compared to the normal control mice. As shown in Table 6, serum biochemical examinations showed reduced GLU levels in the group administered 50 mg/kg/day MO extract compared to the normal control group. We also detected significantly decreased GOT levels in mice administered MO extract at doses of 50 and 450 mg/kg/day compared to mice in the normal control group. Other parameters did not exhibit any significant differences between the MO extract-treated and normal control groups.

## 4. Discussion

Numerous traditional medicines are currently widely used worldwide to treat various ailments; however, they are sometimes associated with potential risks. Therefore, toxicological assessment of these medicines is essential to evaluate their safety, determine suitable doses, and evaluate their side-effects [8]. Moreover, with the increasing demand for insect-based drugs in traditional medicine and their associated toxicity, the safety and efficacy of drugs have become a major public health concern [9]. Toxic components can limit the pharmacological activity of drugs. Additionally, excessive or prolonged exposure to drugs could result in definite damage to organs. In the present study, we evaluated the safety of MO water extract using a standard toxicological study design to assess the potential oral dose toxicity. During the study period of 2 weeks, male C57BL/6 mice were orally administered once daily with doses of 0, 50, 150, or 450 mg/kg/day of MO extract after which several study parameters of mortality, clinical signs, changes in body weight, gross findings, organ weight, histopathological examinations, and hematology were assessed.

Changes in body weight have been previously reported to be linked to the adverse effects of drugs [10]. We observed a similar increase in body weight among MO extract-treated and control mice. In addition, mice treated with the MO extract at 50, 150, and 450 mg/kg/day did not show mortality and clinical signs throughout the study, similar to the control group. These findings indicate that oral administration of repeated doses of the MO extract did not exert any toxicity on the growth and function of mice.

It is well known that a change in the organ weight is a sensitive indicator of the potential toxicity of chemicals [11]. As described above, repeated oral dosing with the MO extract had no effect on the body weight gain in mice administered ≤ 450 mg/kg/day MO extract.

Similar to the body weight, we did not observe any change in the absolute weight of organs, such as the lungs, liver, spleen, kidneys, and testis in mice treated with MO extract. Moreover, no corresponding histopathological changes or accompanying biochemical alterations were detected. Therefore, we concluded that MO extract-mediated changes in weight of these organs are toxicologically insignificant.

The hematopoietic system serves as one of the most important indicators for estimating the toxicity of drugs. Moreover, it is regarded as a critical index of the physiological and pathological status of humans and animals [12] because it reflects the status of bone marrow activity and intravascular effects such as hemolysis and anemia [13]. Therefore, in the present study we assessed the effect of MO extract on various hematological parameters and observed that at repeated doses of up to 450 mg/kg/day MO extract did not cause any hematological abnormalities in the tested mice.

Results of the present 2-week repeated oral dose toxicity study clearly revealed that administration of MO extract did not exert any adverse effect on male mice. Although the correct LD_50_ dose of MO extract remains unclear, the present study provided evidence that acute exposure to different doses of MO extract did not result in any significant toxic effects on male mice, suggesting that the oral LD_50_ of MO extract was greater than 450 mg/kg/day in male mice.

## 5. Conclusion

The extract of MO has traditionally been used as a medicine in several Asian countries, including Korea and China. However, its safety or toxicity has not been reported. In conclusion, a 14-day repeated oral dosing of MO extract to mice resulted in a significant alteration in clinical signs, body weight, and hematological, biochemical, and histopathological parameters at doses of ≤ 450 mg/kg/day. Although we could not confirm the toxic dose of MO extract, the present study is the first of its kind to confirm the toxicity of MO extract.

## Figures and Tables

**Table 1:** Mortality in male mice after 2 weeks of repeated oral administration of MO.

Group (mg/kg/day)	Days after treatment													
	1	2	3	4	5	6	7	8	9	10	11	12	13	14
0	^*∗*^0/5	0/5	0/5	0/5	0/5	0/5	0/5	0/5	0/5	0/5	0/5	0/5	0/5	0/5
50	0/5	0/5	0/5	0/5	0/5	0/5	0/5	0/5	0/5	0/5	0/5	0/5	0/5	0/5
150	0/5	0/5	0/5	0/5	0/5	0/5	0/5	0/5	0/5	0/5	0/5	0/5	0/5	0/5
450	0/5	0/5	0/5	0/5	0/5	0/5	0/5	0/5	0/5	0/5	0/5	0/5	0/5	0/5

*∗*Number of dead mice/total mice.

**Table 2:** Clinical signs in male mice after 2 weeks of repeated oral administration of MO.

Group (mg/kg/day)	Days after treatment													
	1	2	3	4	5	6	7	8	9	10	11	12	13	14
0	^*∗*^0/5	0/5	0/5	0/5	0/5	0/5	0/5	0/5	0/5	0/5	0/5	0/5	0/5	0/5
50	0/5	0/5	0/5	0/5	0/5	0/5	0/5	0/5	0/5	0/5	0/5	0/5	0/5	0/5
150	0/5	0/5	0/5	0/5	0/5	0/5	0/5	0/5	0/5	0/5	0/5	0/5	0/5	0/5
450	0/5	0/5	0/5	0/5	0/5	0/5	0/5	0/5	0/5	0/5	0/5	0/5	0/5	0/5

*∗*Number of mice with clinical signs/total mice.

**Table 3:** Gross findings in male mice after 2 weeks of repeated oral administration of MO.

Group (mg/kg/day)	Gross finding	Frequency
0	No gross findings	5/5
50	No gross findings	5/5
150	No gross findings	5/5
450	No gross findings	5/5

**Table 4:** Organ weights in male mice after 2 weeks of repeated oral administration of MO.

Parameters (g)	Group (mg/kg/day)			
	0	50	150	450
Lung	0.137 ± 0.004	0.134 ± 0.018	0.128 ± 0.008	0.134 ± 0.021
Liver	1.266 ± 0.063	1.228 ± 0.101	1.276 ± 0.083	1.244 ± 0.140
Spleen	0.061 ± 0.017	0.057 ± 0.016	0.056 ± 0.017	0.050 ± 0.007
Kidney	0.321 ± 0.017	0.294 ± 0.024	0.296 ± 0.036	0.300 ± 0.021
Testis	0.178 ± 0.011	0.172 ± 0.019	0.170 ± 0.031	0.174 ± 0.009

Values are expressed as means ± SD of five mice at sacrifice.

**Table 5:** Hematological values in male mice after 2 weeks of repeated oral administration of MO.

Parameters	Group (mg/kg/day)			
	0	50	150	450
RBC (× 10^6^/*μ*g)	8.480 ±1.182	7.400 ± 2.224	8.680 ± 2.366	9.620 ± 0.554
HB (g/dL)	14.600 ±1.140	10.200 ± 3.347	12.600 ± 3.782	14.400 ± 0.894
HCT (%)	42.100 ± 11.520	36.480 ± 12.156	42.640 ± 12.182	47.680 ± 2.310
MCV (fl)	49.420 ± 1.099	48.820 ± 2.922	48.940 ± 1.122	49.640 ± 1.016
MCH (pg)	14.800 ± 0.447	14.200 ± 0.837	14.400 ± 0.894	14.800 ± 0.447
MCHC (g/dL)	29.880 ± 0.879	28.200 ± 2.021	28.860 ± 1.252	29.620 ± 0.610
PLT (× 10^3^/*μ*g)	729.400 ± 122.100	685.200 ± 249.905	661.000 ± 83.382	715.800 ± 65.370
WBC (× 10^3^/*μ*g)	2.240 ± 1.314	2.720 ± 1.224	2.320 ± 1.274	3.160 ± 1.383
NEU (%)	15.320 ± 9.066	10.200 ± 1.495	17.360 ± 8.914	21.180 ± 15.790
LYM (%)	79.140 ± 11.305	84.960 ± 3.087	76.440 ± 9.095	74.360 ± 17.568
MON (%)	3.300 ± 0.846	3.280 ± 1.152	3.440 ± 1.410	2.960 ± 1.601
EOS (%)	1.640 ± 2.263	1.100 ± 1.177	1.860 ±1.633	1.040 ± 1.488
BAS (%)	0.620 ± 0.567	0.480 ± 0.634	0.900 ± 0.552	0.460 ± 0.650

RBC, red blood cells; HB, hemoglobin; HCT, hematocrit; MCV, mean corpuscular volume; MCH, mean corpuscular hemoglobin;

MCHC, mean corpuscular hemoglobin concentration; PLT, platelet; WBC, white blood cell; NEU, neutrophil; LYM, lymphocyte;

MON, monocyte; EOS, eosinophil; BAS, basophil.

Values are expressed as means ± SD of five mice at sacrifice.

**Table 6:** Serum biochemical values in male mice after 2 weeks of repeated oral administration of MO.

Parameters	Group (mg/kg/day)			
	0	50	150	450
CRE (mg/dL)	1.620 ± 0.228	1.320 ± 0.259	1.560 ± 0.451	1.600 ± 0.678
GLU (mg/dL)	172.000 ± 6.218	152.500 ± 12.793^*∗*^	159.750 ± 29.113	161.250 ± 33.925
GOT (U/L)	85.000 ± 5.354	72.500 ± 8.515^*∗*^	81.500 ± 15.000	72.750 ± 4.646^*∗*^
GPT (U/L)	55.000 ± 9.566	53.800 ± 9.039	64.400 ± 37.226	52.000 ± 12.268
BUN (mg/dL)	25.675 ± 2.712	24.920 ± 2.543	24.240 ± 2.237	25.780 ± 2.160
TBIL (mg/dL)	0.725 ± 0.222	0.900 ± 0.082	1.050 ± 0.173	1.025 ± 0.126

CRE, creatinine; GLU, glucose; GOT, glutamic oxaloacetic transaminase; GPT, glutamic pyruvic transaminase;

BUN, blood urea nitrogen; TBIL, total bilirubin.

Values are expressed as means ± SD of five mice at sacrifice.

*∗*Significantly different from the normal control at p < 0.05.

## Data Availability

The data used to support the findings of this study are available from the corresponding author upon request.
